# The effects of phosphatidic acid supplementation on strength, body composition, muscular endurance, power, agility, and vertical jump in resistance trained men

**DOI:** 10.1186/s12970-016-0135-x

**Published:** 2016-06-02

**Authors:** Guillermo Escalante, Michelle Alencar, Bryan Haddock, Phillip Harvey

**Affiliations:** California State University- San Bernardino, 5500 University Parkway, San Bernardino, CA 92407 USA; California State University- Long Beach, 1250 Bellflower Boulevard, Long Beach, CA 90840 USA; Max Muscle Sports Nutrition, 210 West Taft Avenue, Orange, CA 92865 USA; University of Phoenix, San Diego Campus, 9645 Granite Ridge Drive, San Diego, CA 92123 USA

**Keywords:** Phospholipid, Muscle protein synthesis, Hypertrophy, Lean body mass, Fat mass

## Abstract

**Background:**

Phosphatidic acid (PA) is a lipid messenger that has been shown to increase muscle protein synthesis via signaling stimulation of the mammalian target of rapamycin (mTOR). MaxxTOR® (MT) is a supplement that contains PA as the main active ingredient but also contains other synergistic mTOR signaling substances including L-Leucine, Beta-Hydroxy-Beta-Methylbutyrate (HMB), and Vitamin D3.

**Methods:**

Eighteen healthy strength-trained males were randomly assigned to a group that either consumed MT (*n* = 8, 22.0 +/− 2.5 years; 175.8 +/− 11.5 cm; 80.3 +/− 15.1 kg) or a placebo (PLA) (*n* = 10, 25.6 +/− 4.2 years; 174.8 +/− 9.0 cm; 88.6 +/− 16.6 kg) as part of a double-blind, placebo controlled pre/post experimental design. All participants volunteered to complete the three day per week resistance training protocol for the eight week study duration. To determine the effects of MT, participants were tested on one repetition maximum (1RM) leg press strength (LP), 1RM bench press strength (BP), push-ups to failure (PU), vertical jump (VJ), pro-agility shuttle time (AG), peak power output (P), lean body mass (LBM), fat mass (FM), and thigh muscle mass (TMM). Subjects were placed and monitored on an isocaloric diet consisting of 25 protein, 50 carbohydrates, and 25 % fat by a registered dietitian. Separate two-way mixed factorial repeated measures ANOVA’s (time [Pre, Post] x group [MT and PLA] were used to investigate strength, body composition, and other performance changes. Post-hoc tests were applied as appropriate. Analysis were performed via SPSS with significance at (p ≤ 0.05).

**Results:**

There was a significant main effect (F_(1,16)_ = 33.30, *p* < 0.001) for LBM where MT significantly increased LBM when compared to the PLA group (*p* < 0.001). Additionally, there was a significant main effect for LP (F_(1,16)_ = 666.74, *p* < 0.001) and BP (F_(1,16)_ = 126.36, *p* < 0.001) where both increased significantly more in MT than PLA group (*p* < 0.001). No significant differences between MT and PLA were noted for FM, TMM, VJ, AG, P, or PU.

**Conclusion:**

The results of this eight week trial suggest that the addition of MaxxTOR® to a 3-day per week resistance training program can positively impact LBM and strength beyond the results found with exercise alone.

## Background

Resistance training has been proven to help increase or maintain muscle mass as well as strength for various populations [1]. In an effort to safely maximize the effects of a resistance training program, researchers have investigated the effectiveness of utilizing sports supplements. Sports supplements such as creatine, branched-chain amino acids, and whey protein are among the most commonly researched topics in sports nutrition targeted to improve muscle mass, strength, and/or sports performance [2].

Phospholipids are another class of sports supplement that have been studied for their effect on athletic performance [3]. Phosphatidic acid (PA) is a phospholipid that makes up a small percentage of the phospholipid pool and is a compound formed by two fatty acids and a phosphate group that are covalently bonded to a glycerol molecule through ester linkages [4, 5]. PA is a precursor for the production of other lipids, it can act as a signaling lipid, and it is a major component of cell membranes. Recent research findings have demonstrated a link between PA and muscle protein synthesis [6–9]. A protein kinase known as the mammalian target of rapamycin (mTOR) has been recognized as a critical regulator of muscle protein synthesis [10–16]. Research findings have demonstrated that elevations in amino acids [10, 11], growth factors [12, 13], and energy status [14–16] can increase muscle protein synthesis through an mTOR dependent mechanism. While research has indicated that PA plays a critical role in mTOR signaling, the exact mechanism by which PA stimulates mTOR has not been confirmed. However, it is hypothesized that PA primarily works via direct binding to mTOR [6, 7].

Recent studies demonstrate that a mechanical stimulus, such as resistance training or passively stretching skeletal muscles, can create an increase in the intracellular levels of PA and that an increase in PA contributes to the activation of mTOR-dependent signaling events [7, 8]. It has also been demonstrated that exogenous sources of PA can promote the activation of mTOR signaling [9]. The results of these studies suggest that both mechanical stimuli and the exogenous addition of PA can stimulate mTOR signaling through different pathways that collectively may contribute to a larger effect of mTOR signaling.

Although there has been a significant amount of research performed at the molecular level on the effects of PA on mTOR signaling, more research is needed to fully confirm that mTOR signaling may be stimulated by PA. Conversely, only two studies have investigated the effects of PA supplementation with a resistance training program on human performance. Research performed by Hoffman et al. [17] investigated the efficacy of PA ingestion on lean body mass (LBM), muscle thickness, and strength in resistance trained men. The authors concluded that the combination of ingesting 750 mg of PA daily during a resistance training program appear to increase strength and lean body mass more than those not taking the PA. Despite these positive findings, two weaknesses of this study were that the subjects were not supervised in their resistance training program and diet was not overseen throughout the study. A more recent study performed by Joy et al. [18] concluded that PA supplementation, as compared to receiving a placebo (PLA), led to significantly increased skeletal muscle hypertrophy, LBM, and maximal strength following a supervised 8-week resistance training program and a customized diet plan.

MT is a dietary supplement that contains 750 mg of PA as the main active ingredient but also contains other synergistic ingredients including L-Leucine, Beta-Hydroxy-Beta-Methylbutyrate (HMB) and Vitamin D3 to deliver mTOR signaling activation. L-Leucine is a branched-chain amino acid that has been shown to have the highest anabolic effect compared to other amino acids on activating protein synthesis and muscle cell growth while decreasing the rate of protein degradation in muscles [19–22]. L-Leucine has also been suggested to have a sparing effect on muscle glycogen and can lead to decreases in proton production [19–22]. HMB is a metabolite of L-Leucine and numerous studies have demonstrated that HMB supplementation combined with resistance training can increase protein synthesis, strength, and lean body mass [20, 23, 24]. It has been suggested that HMB can increase protein synthesis by supporting the integrity of muscle fibers, protecting critical contractile proteins, and improving recovery by attenuating exercise-induced damage in trained and untrained subjects [20, 23, 24]. Despite the positive results reported on the efficacy of HMB, there have also been some contradictory studies [25, 26]. In a review of HMB on exercise performance and body composition across varying levels of age, sex, and training experience, Wilson et al. [27] concluded that conflicting results may be attributed to the variability in humans, inadequate sample sizes, and methodological issues such as the specificity of testing conditions, cases of overtraining, an inadequate training stimulus in experienced participants, limited dependent variables, and short duration experiments. Furthermore, vitamin D3 has also been shown to play an important role on muscle mass and function by enhancing the stimulating effect of L-Leucine and insulin on protein synthesis [28].

The overall purpose of this investigation was to study the effects of the dietary supplement MT in conjunction with a 3-day per week total body resistance training program on muscular strength, muscular endurance, power(P), vertical jump (VJ), agility (AG), lean body mass (LBM), thigh muscle mass (TMM), and fat mass (FM) in resistance trained men.

## Methods

### Subjects

Initially, nineteen healthy, strength-trained male volunteers signed an informed consent form approved by the Institutional Review Board at California State University, San Bernardino and agreed to participate in this randomized, double-blind, placebo-controlled study. One subject voluntarily withdrew from the study due to time constraints. Eighteen participants completed the trial, where ten (25.6 ± 4.2 years, 174.8 ± 9.0 cm, 88.6 ± 16.6 kg) were randomly assigned to the PLA group and eight (22.0 ± 2.5 years, 175.8 ± 11.5 cm, 80.3 ± 15.1 kg) were randomly assigned to the MT group. The protocol and subject inclusion/exclusion criteria were similar to the study performed by Joy et al. (18).

All participants were required to abstain from consuming any muscle-building supplements (e.g., creatine) for at least 1 month prior to pretest measures, abstain from training outside of the prescribed protocol during the study, be non-smokers, have resistance training experience of no less than one year, and have participated in resistance training at least three days per week for the past six months to be included in this study. Additionally, participants had to be free of any injuries or medical conditions that would prohibit them from participating in a resistance training program. Measures of 1 repetition maximum (1RM) LP, 1RM BP, LBM, FM, TMM, PU, AG, VJ, and P were taken within 7 days prior to, and within 7 days following, the resistance training/supplementation protocol. All resistance training sessions were supervised by certified personal trainers and took place three days per week with 48–72 h between resistance training sessions. Each body part was trained 1–2 times per week following a daily undulating periodization protocol (Table 1). Each participant performed a 5RM for each exercise prior to the first four weeks with the exception of the BP and LP, in which true 1RM values were determined. Five repetition maximum testing was repeated at the end of week 4 for the new exercises.

**Table 1:** Eight-week resistance training protocol

Monday		Wednesday		Friday						
Week 1–4	Week 5–8	Week 1–4	Week 5–8	Week 1–4	Week 5–8		Monday & Wednesday Repetitions	Friday Repetitions	Monday & Wednesday Rest	Friday Rest
Leg Press	Leg Press	Bent Over Row	Pendlay Rows	Leg Press	Leg Press	Week 1	12	5	45 s	3–5 m
Leg Extension	Safety Bar Squat	Barbell Shrug	Hexbar Shrug	Bench Press	Bench Press	Week 2	10	3	60 s	3–5 m
Leg Curl	Barbell Lunge	Straight Arm Pull Down	Pulldown	Leg Extension	Safety Bar Squat	Week 3	8	2	90 s	3–5 m
Hyperextension	Stiff Leg Deadlift	Australian Row	Decline DB Row	Close Grip Press	Flat DB Press	Week 4	6	1	120 s	3–5 m
Bench Press	Bench Press	Barbell Shoulder Press	DB Shoulder Press			Week 5	12	5	60 s	3–5 m
Incline DB Press	Flat DB Press	Isolated Barbell Military	Upright Row			Week 6	10	3	60 s	3–5 m
Close Grip Bench Press	Cable Cross Over	DB Lateral Raise	Barbell Front Raise			Week 7	8	2	90 s	3–5 m
Cable Rope Extensions	Skull Crusher	DB Bicep Curls	Barbell Bicep Curl			Week 8	6	1	120 s	3-5 m

### Strength testing and training

Strength testing and training followed the protocol performed by Joy et al. (18). The 1-RM testing protocol consisted of 1 set of 10–12 repetitions at approximately 50 % 1-RM followed by 1 set of 2–3 repetitions at approximate intensities of 75 and 85 % 1RM. After the final warm-up set, weight was increased in 5–20 lb increments until a 1-RM was attained. Five repetition maximum testing followed an identical pattern; however, intensities were relative to a 5-RM instead of a 1-RM. These RM values were used to calculate the load used for each exercise for each participant. Training exercises (Table 1) were altered at week 5 to introduce a more novel stimulus. All participants were required to perform a set number of repetitions with their prescribed training load. In the event that a subject reached muscular failure, a certified personal trainer assisted with the completion of the exercise. Strength was assessed via 1RM testing of the LP and BP. In order for a repetition to count as a successful attempt in the leg press, the participant had to reach an angle of 90° at their knee joints as agreed upon unanimously by 3 trained research assistants. In order for a repetition to count as a successful attempt in the bench press, the participant had to touch the barbell to their chest at the bottom of the movement and lock out their arms at the top of the movement as agreed upon unanimously by 3 trained research assistants. Testing for LP and BP strength took place on one day for each subject.

### Lean body mass, Fat mass, and thigh muscle mass assessment

A total body DXA (Prodigy™; Lunar Corporation, Madison, WI) scan was performed to measure total body composition as described by Maden-Wilkinsen et al. [29]. Participants laid supine on the scanning bed. The GE Lunar Prodigy computer software was used to provide estimations of total LBM and FM [29]. After total LBM and FM were computed and documented, each participant’s dominant thigh was identified as a region of interest using previously reported borders from the femoral neck to the knee joint [29] to determine TMM. Lean mass, FM, and bone mineral content were estimated from the selected region of interest. All DXA analyses were performed by a single certified technician and quality assurance was performed per the manufacturer instructions. In estimating lean mass the typical DXA machine includes not just muscle mass but also connective tissue and the non-mineral components of bone [29]. Bone mineral content (BMC) accounts for approximately 55 % of total bone mass with the rest being made up by protein and water [29]. For this reason, an adjusted lean mass was calculated as follows [29]: Lean mass = total mass - fat mass - (1.82 * BMC). DXA also includes non-adipose components of fat tissue, such as protein, in the lean mass but the contribution this makes is unclear. Hence, no further adjustments were applied [29]. Thigh muscle mass hypertrophy was determined via changes in adjusted thigh lean mass of the dominant thigh as reported by the DXA. Testing for body composition and TMM took place prior to strength testing on the same day.

### Vertical jump assessment

Vertical jump (VJ) was assessed by using a Vertec (Vertec2, Sports Imports, Columbus, OH, USA). The participant’s reach height was measured and recorded by first adjusting the height of the Vertec plastic vanes to be within the subject’s reach [30]. The participant stood directly beneath the Vertec and reached as high as possible with their dominant hand without lifting the heels from the floor and touched the highest vane possible [30]. After initial familiarization with the procedures and the Vertec apparatus, the subject warmed-up at a self-selected resistance on a stationary bicycle for five minutes and was allowed to perform several trials of the counter jump procedure with the Vertec [30]. The subjects were instructed to not take any lead-up steps prior to the jump but were allowed to perform a rapid countermovement by quickly descending into a squat while swinging the arms down and back [30]. The rapid countermovement was immediately followed by a maximal jump in which the dominant hand reached to touch the highest possible Vertec vane [30]. Three trials were allowed and the highest was recorded [30]. The vertical jump height recorded was the difference between the highest jump and the reach height [30]. Testing for the VJ took place the day prior to strength testing.

### Agility assessment

Agility (AG) was assessed with the pro-agility shuttle [30]. Timing gates (Swift Performance, Speedlight V2 wireless timing system, Walco, Australia) were set up five yards apart and the participant started from the middle timing gate; an upright stance was used and the subject faced forward [30]. The timing gate device randomly sent out a beep to signal the subject to start the agility assessment while simultaneously starting the timer. Upon hearing the beep, the subject turned to the left and sprinted for 5 yards; then turned to the right and sprinted for 10 yards and finally turned back to the left and sprinted for five yards back to the starting point [30]. The lines marking the distance of the timing gates, which were set up with athletic tape on the floor, had to be contacted by each foot [30]. Three trials were performed for the pre-test and post-test and the fastest time was recorded; three minutes of rest was provided between each trial [30]. Testing for AG took place approximately three minutes after VJ assessment was performed.

### Muscular endurance

Muscular endurance was assessed with the subject performing standard push-ups (PU) to failure the feet on the floor and the hands shoulder width apart [30]. The starting position was the bottom position of the push-up with the area of the body from the chest to the thighs making contact with the floor [30]. The participant pushed themselves up from the bottom position with the body straight such that a line could be drawn from the shoulder joint to the ankle joint [30]. The participant then lowered their body back to the starting position and repeated the pattern [30]. The participant continued to exercise at a comfortable rate of 20–30 repetitions per minute until no more PU could be performed with correct form [30]. A push up was counted by a trained research assistant when the participant was in the up position; no resting was allowed between repetitions [30]. Testing for muscular endurance took place approximately three minutes after AG assessment was performed.

### Power assessment

Power (P) output was recorded in real time with a Monark cycle ergometer (Monark model 828E, Vansbro, Sweden) that was connected to the Monark Anaerobic test software (Monark 828E Analysis Software 3.0, Monark, Vansbro, Sweden). During the cycling test, the participant was instructed to cycle against a predetermined resistance (7.5 % of body weight) as fast as possible for 10 s [31]. The saddle height was adjusted to the individual’s height to produce approximately 10° knee flexion while the foot was in the low position of the central void. A standardized verbal stimulus was provided to the subject. Power output testing took place approximately three minutes after muscular endurance was assessed.

### Dietary/Supplement supervision

Prior to the study, participants were required to watch a video made by a registered dietitian specializing in sports nutrition discussing their diet protocols, the diet recording/reporting protocol, and emphasizing the importance of adherence to the diet plan. Two weeks prior to the start of training each participant was provided with an individual meal plan, designed by the dietitian. This meal plan was to be followed throughout the study. Daily caloric need for each participant was estimated via the Harris Benedict equation and was designed to be iso-caloric in nature by adding 55 % more calories to their resting metabolic rate estimation in order to compensate for their moderate activity level of strength training three days per week. The diet consisted of 25 % protein, 50 % carbohydrates, and 25 % fat. Although a sample meal plan was provided for each subject, the dietitian explained in the video that participants could choose any foods they desired as long as the final calorie count and macronutrient breakdown was within the guidelines provided. After the video was watched by the participants, the registered dietitian and principal investigator oversaw the diet logs of the participants throughout the study. All participants were instructed to use the smartphone app MyFitnessPal® to record their nutritional intake and to submit a weekly summary of their diet logs from the MyFitnessPal® website via an email to ensure compliance. Subjects not familiar with the mobile app were instructed by the research team on how to utilize it, but twelve of the eighteen subjects had used this app prior to this study. The use of mobile apps for dietary self-reporting has been previously used in research [32]. The MyFitnessPal® app is a database comprised of over 5 million foods that have been provided by users via entering data manually or by scanning the bar code on packaged goods. Thus, the data themselves are primarily derived from food labels (i.e., Nutrition Facts Panel) derived from the USDA National Nutrient database. The breakdown of dietary intake over the course of the study can be found in the results section.

In addition to the recommended food intake, the MT group received 5 capsules of MT per day per day while the PLA group received 750 mg of rice flour, each delivered in 5 visually identical capsules. A single production lot of both MT and PLA supplements were manufactured in a facility compliant with current Good Manufacturing Practices (cGMP) for dietary supplements (21 CFR 111). Phosphatidic acid purity and potency were analyzed by an ultra-performance liquid chromatograph with triple quadrupole mass spectrometry (LC/MS/MS) methods (Avanti Polar Lipids, Inc., Alabaster, AL). Purity and potency of L-Leucine, HMB and vitamin D3 were analyzed by High Pressure Liquid Chromatography (HPLC) at Micro Quality Laboratories, Inc, Burbank, CA. On resistance training days, participants consumed 3 capsules of their respective supplement 30 min prior to resistance training and 2 capsules immediately following resistance training along with 24 g of hydrolyzed collagen protein powder from beef skin (Peptiplus XB agglomerated, Gelita AG, Eberbach, Germany) mixed with 500 ml water. The protein supplement was provided by the researchers in order to ensure control for post-exercise meals between groups. Furthermore, hydrolyzed collagen protein was chosen as it is an incomplete protein source low in leucine in order to potentially minimize the impact the supplement being studied. On non-resistance training days, participants consumed 3 of their respective supplement pills with breakfast and 2 pills with dinner. In order to ensure compliance, participants were required to return to the laboratory with their empty containers 3 weeks after starting the study in order to receive their next bottle of supplements. Three weeks later, they were required to return to the laboratory again before they received their last bottle of the supplement. Since the dietician/principal investigator had weekly interaction with the participants via email, all participants were monitored for compliance throughout the study. Each subject turned in empty bottles at the designated times and reported taking the supplement as directed.

Product formulations were blinded and coded to both the investigators and the participants so that neither knew which formulation was consumed during the study. Each participant randomly selected a 4 digit participant code that corresponded to a code on their respective bottles. The research team recorded the code on each bottle for each participant; however, the key for each code that determined whether the bottle contained MT or PLA was revealed to the researchers after all the data was collected at the end of the study. Each participant was provided with their supplement bottle (PLA or MT) for 3 weeks on the first Monday and every third Monday thereafter.

### Statistical analysis

Separate two-way mixed factorial Repeated Measures Analysis of Variance (time [Pre, Post] x group [MT and PLA] were used to investigate body composition changes (LBM, FM, TMM), strength changes (BP, LP), and other performance changes (PU, P, VJ, and AG). When significant main effects were found, a Tukey post-hoc was conducted to determine where the differences occurred. Data is presented as mean ± standard deviation. All analyses were performed using SPSS version 22 (SPSS, Inc., Chicago, IL). An alpha level was set at *p* ≤ 0.05.

## Results

**Fig. 1:**
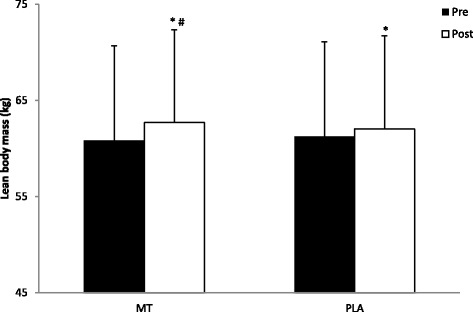
Changes in Lean Body Mass. All data are reported as mean +/− SD (*denotes significantly different from pre, # denotes significantly different from PLA)

**Fig. 2:**
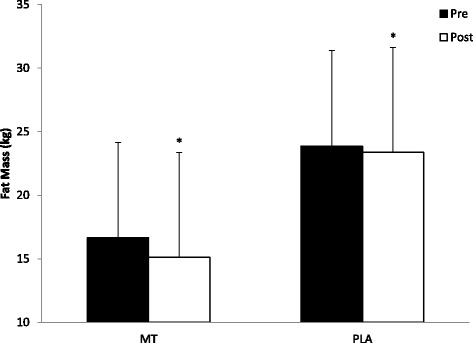
Changes in Fat Mass. All data are reported as mean +/− SD (*denotes significantly different from pre, # denotes significantly different from PLA)

**Fig. 3:**
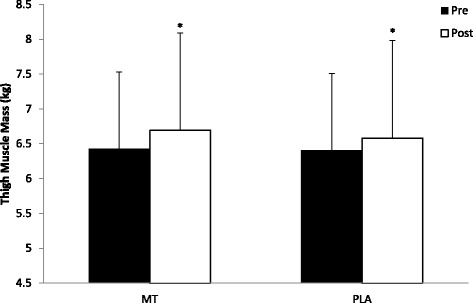
Changes in Thigh Muscle Mass. All data are reported as mean +/− SD (*denotes significantly different from pre, # denotes significantly different from PLA)

There were no significant differences between the MT or the PLA group for any baseline measurement (Table 2). The weekly nutrition logs turned in by the participants to the principal investigator on a weekly basis throughout the study also showed no significant differences in calories consumed (MT: 2709.0 +/− 357.0 Cal vs PLA: 2681.7 +/− 277.8 Cal), carbohydrates consumed (MT: 320.4 +/− 47.8 g vs PLA: 325.9 +/− 34.3 g), protein consumed (MT: 166.5 +/− 22.9 g vs PLA: 158.0 +/− 27.8 g), and fat consumed (MT: 84.7 +/− 26.5 g vs PLA: 82.9 +/− 14.3 g). There was a significant group x time interaction for LBM (F_(1,16)_ = 33.30, *p* = 0.041) where the MT group increased LBM to a greater extent (pre: 60.8 ± 9.5 kg; post: 62.7 ± 10.2 kg) when compared to the PLA group (pre: 61.2 ± 9.7 kg; post: 62.0 ± 9.7 kg) (Fig. 1). There was a significant time effect for FM (F_(1,16)_ = 8.64, *p* = 0.010) where the MT group (pre: 16.6 ± 7.2 kg; post: 15.1 ± 7.8 kg) tended to lose more FM than the PLA group (pre: 23.9 ± 8.1 kg; post: 23.4 ± 8.3 kg) (Fig. 2). Furthermore, there was a significant time effect for TMM (F_(1,16)_ = 5.652, *p* = 0.030) where the MT group (pre: 6.43 ± 1.10 kg; post: 6.69 ± 1.13 kg) and the PLA group (pre: 6.41 ± 1.45 kg; post: 6.58 ± 1.35 kg) both gained TMM (Fig. 3). Pre versus post individual changes in body weight, body fat percentage, LBM, and FM further illustrate the changes observed in body composition over the course of the study (Table 3).

**Table 2:** Body composition, strength, and performance baseline measurements

Variable	MT group	PLA group
Lean Body Mass (LBM)	60.8 ± 9.5 kg	61.2 ± 9.7 kg
Fat Mass (FM)	16.6 ± 7.2 kg	23.9 ± 8.1 kg
Thigh Muscle Mass (TMM)	6.43 ± 1.10 kg	6.41 ± 1.45 kg
1-RM Leg Press (LP)	292.6 ± 60.5 kg	306.8 ± 62. 5 kg
1-RM Bench Press (BP)	92.0 ± 18.1 kg	98.6 ± 26.5 kg
Power (P)	897.6 ± 178.3 W	854.3 ± 238.7 W
Agility (AG)	5.16 ± 0.34 s	5.45 ± 0.35 s
Vertical Jump (VJ)	62.6 ± 11.1 cm	56.6 ± 10.2 cm
Push-Ups (PU)	31.9 ± 7.6	30 ± 7.2

Data presented as Mean +/− SD. *n* = 8 for MT and *n* = 10 for PLA

**Table 3:** Individual changes in body weight, body fat percentage, LBM and FM

	Bodyweight (Kg)		Body Fat %		LBM (Kg)		FM (Kg)	
	Pre	Post	Pre	Post	Pre	Post	Pre	Post
MT								
1	74.6	74.6	24.2	20.9	54.1	56.7	17.3	15.0
2	80.0	80.1	19.1	16.9	62.0	63.6	14.6	12.9
3	60.1	59.6	19.8	18.7	45.8	46.5	11.3	10.7
4	111.3	113.6	30.8	30.2	75.4	77.2	33.6	33.3
5	86.5	82.5	20.6	15.3	66.3	67.2	17.2	12.2
6	70.0	69.6	17.1	12.5	56.2	58.1	11.6	8.3
7	75.0	77.7	21.1	23	56.7	57.4	15.1	17.1
8	85.30	88.6	15	13.2	70.0	74.7	12.4	11.4
MT Mean +/− SD	80.3 +/− 15.1	80.8 +/− 15.9	21.0 +/− 4.8	18.8 +/− 5.8	60.8 +/− 9.5	62.7 +/− 10.2	16.6 +/− 7.2	15.1 +/− 7.8
PLA								
1	93.4	93.6	27.1	25.2	65.3	67.2	24.3	22.6
2	78.1	80.0	26.7	26.4	55.2	56.8	20.1	20.4
3	70.0	69.6	24.3	23.6	51.0	51.1	16.4	15.8
4	81.4	81.8	27.9	27.0	56.7	57.7	21.9	21.4
5	69.6	70.0	26.9	26.0	49.1	50.1	18.1	17.6
6	108.9	110.0	34.4	34.1	68.5	69.8	36.1	36.1
7	86.4	85.9	20.1	19.6	66.0	66.0	16.6	16.1
8	80.2	79.1	21.6	20.2	59.7	59.9	16.4	15.2
9	120.0	120.5	29.1	28.5	81.9	82.6	33.1	32.9
10	98.6	99.1	37.8	37.5	58.7	59.3	35.7	35.6
PLA Mean +/− SD	88.6 +/− 16.6	89.0 +/− 16.8	27.6 +/− 5.3	26.8 +/− 5.6	61.2 +/− 9.7	62.0 +/− 9.7	23.9 +/− 8.1	23.4 +/− 8.3

**Fig. 4:**
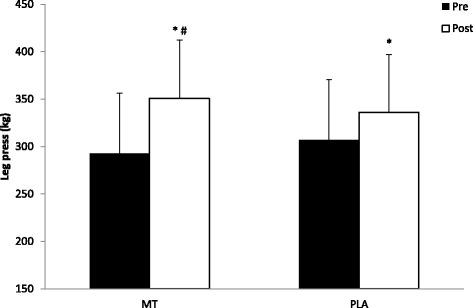
Changes in Leg Press Strength. All data are reported as mean +/− SD (*denotes significantly different from pre, # denotes significantly different from PLA)

**Fig. 5:**
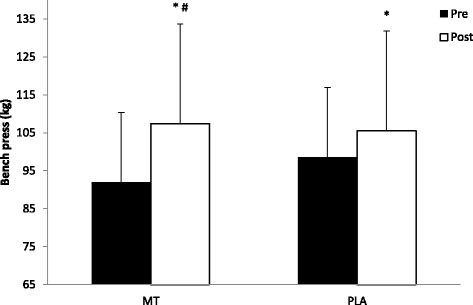
Changes in Bench Press Strength. All data are reported as mean +/− SD (*denotes significantly different from pre, # denotes significantly different from PLA)

Pre to post changes in muscle strength were seen in both 1-RM LP and 1-RM BP. There was a significant group x time interaction (F_(1,16)_ = 74.28, *p* < 0.001) for 1-RM LP where the MT group increased leg strength significantly greater (pre: 292.6 ± 60.5 kg; post: 350. 9 ± 66.9 kg) than the PLA group (pre: 306.8 ± 62. 5 kg; post: 335.9 ± 59.9 kg). Similarly, there was a significant group x time interaction (F_(1,16)_ = 18.69, *p* < 0.001) for the BP where the MT group increased 1-RM BP significantly greater (pre: 92.0 ± 18.1 kg; post: 107.4 ± 18.6 kg) than the PLA group (pre: 98.6 ± 26.5 kg; post: 105. 5 ± 26.1 kg). Strength changes for the LP and BP can be seen in Figs. 4 and 5, respectively.

Pre to post changes in performance measures were seen between the MT and PLA groups. There was a significant effect for time (F_(1,16)_ = 43.96, *p* < 0.001) for PU in the MT group (pre: 31.9 ± 7.6; post: 37.4 ± 7.4) and PLA group (pre: 30 ± 7.2; post 34.8 ± 6.3) where the MT group and the PLA group both performed more PU. Similarly, there was a significant effect for time (F_(1,16)_ = 20.69, *p* < 0.001) for P in the MT group (pre: 897.6 ± 178.3 W; post: 989.5 ± 180.0 W) and the PLA group (pre: 854.3 ± 238.7 W; post: 903.6 ± 231.0 W) where the MT group tended to produce more P than the PLA group. There were no significant interactions for AG or VJ between groups. Performance changes can be seen in Table 4.

**Table 4:** Changes in performance variables

Performance Variable	MT Group		PLA Group	
	PRE	POST	PRE	POST
Power (P)	897.6 ± 178.3 W	989.5 ± 180.0 W^a^	854.3 ± 238.7 W	903.6 ± 231.0 W^a^
Agility (AG)	5.16 +/− 0.34 s	5.03 +/− 0.39	5.45 +/− 0.35 s	5.31 +/− 0.53
Vertical Jump (VJ)	62.6 +/− 11.1 cm	63.4 +/− 8.6 cm	56.6 +/− 10.2 cm	58.0 +/− 7.2 cm
Push-Ups (PU)	31.9 ± 7.6	37.4 ± 7.4^a^	30 ± 7.2	34.8 ± 6.3^a^

Data are presented as mean +/− SD. *n* = 8 for MT and *n* = 10 for PLA (^a^ denotes significant time effect)

## Discussion

The current study investigates the effects of PA on body composition, strength, muscle endurance, AG, P, and VJ in resistance trained males. This study supports the evidence provided by previous researchers [17, 18]. Similar to the studies performed by Hoffman et al. [17] and Joy et al. [18], this investigation provided 750 mg of PA to the experimental group or an identical looking PLA to the PLA group on a daily basis. Also similar to the design of previous researchers [17, 18], each participant received a collagen based protein drink after every workout. In comparison to the first two studies, the supplement (MT) provided to the experimental group in this study had an added proprietary blend of L-Leucine, HMB, and Vitamin D3 to the 750 mg of PA. Furthermore, this investigation examined more fitness parameters including assessments in VJ, AG, and muscular endurance. Table 5 provides a summary of the comparison of the studies on the effects of MT versus PA alone on strength, body composition, thigh hypertrophy, and power.

**Table 5:** Comparison on the effects of MT vs PA alone on strength, body composition, and power

Variable	Current Study (Supplement = MT)		Joy et al. (2014) (Supplement = PA only)		Hoffman et al. (2012) (Supplement = PA only)	
	MT % Change	PLA % Change	PA % Change	PLA % Change	PA % Change	PLA % Change
Bench Strength	16.7 % ↑	7.0 % ↑	7.1 % ↑	5.1 % ↑	5.1 % ↑	3.3 % ↑
Leg Strength	19.7 % ↑	9.1 % ↑	22.7 % ↑	14.3 % ↑	12.7 % ↑	9.3 % ↑
Total Strength	19.1 % ↑	8.7 % ↑	18 % ↑	11.7 % ↑	9.1 % ↑	6.6 % ↑
Lean Body Mass	3.1 % ↑	1.4 % ↑	4.0 % ↑	2.0 % ↑	2.6 % ↑	0.1 % ↑
Fat Mass	9.2 % ↓	2.1 % ↓	8.6 % ↓	3.8 % ↓	0 %	0 %
Thigh Hypertrophy	4.0 % ↑	2.7 % ↑	22.2 % ↑	13.3 % ↑	14.8 % ↑	15.5 % ↑
Power	10.2 % ↑	5.8 % ↑	8.2 % ↑	8.7 % ↑	NA	NA

Data are presented as % change between pre and post for the experimental group and placebo group for each variable

The effects of PA on strength was reported in this study as well as in previous investigations [17, 18]. In comparison to previous studies on PA [17, 18], significant changes in 1-RM BP were only reported in this study. Although Joy et al. [18] also reported an increase in both the PA and PLA groups in 1-RM BP, the observed differences were not significant (*p* = 0.11). Similarly, Hoffman et al. [17] reported that that the magnitude based inferences were unclear regarding any benefit in upper body strength improvements in those participants consuming the PA. Conversely, the effects of PA on leg strength in this study was similar to that reported by previous researchers [17, 18] where all three studies demonstrated an improvement in lower body strength. Although Hoffman et al. [17] reported that the observed changes lacked a significant group x time interaction between the PA and PLA groups (*p* = 0.19) in their investigation, they stated that the magnitude based inferences on changes observed in 1-RM squat suggest a likely benefit from PA on increasing lower body strength. Similarly, all studies demonstrated an increase in total strength, which was defined by Joy et al. [18] as the sum of 1-RM BP and 1-RM leg strength test.

The effects of PA on body composition was also reported in all three investigations. In comparing the effects of PA on LBM between the three studies, positive significant changes were reported in this investigation as well as in the study performed by Joy et al. [18]. Although Hoffman et al. [17] reported a significant main effect (*p* = 0.045) for LBM, the increase in LBM in the PA group was only reported as a trend (*p* = 0.065) towards significant interaction. The effects of PA on FM was also investigated in all three studies; however, it should be noted that none of the investigations were designed to maximize fat loss as evidenced by the exercise prescription as well as the iso-caloric diet the participants followed. The present study and the study performed by Joy et al. [18] both demonstrated a significant time effect for the PA group and the PLA groups, but the investigation performed by Hoffman et al. [17] reported no significant time effect (*p* = 0.95) or group x time interaction (*p* = 0.99) for changes in FM for the PA or PLA groups.

The effects of PA on thigh hypertrophy also varied between this study and that reported by previous researchers [17, 18]. Part of this variability is likely contributed to the different assessment tools and/or methods used to quantify thigh hypertrophy. The study performed by Hoffman et al. [17] used ultrasonography measurements to measure vastus lateralis fascicle thickness by determining the distance between the subcutaneous adipose tissue and intermuscular interface. Although Joy et al. [18] also used an ultrasound device to measure changes in quadriceps hypertrophy, rectus femoris cross sectional area was measured as opposed to vastus lateralis thickness. In this investigation, thigh hypertrophy was measured by determining TMM utilizing a DXA machine where the participant’s dominant thigh region was identified as a region of interest. The current study and the study performed by Hoffman et al. [17] demonstrated only a time effect for both the PA and PLA groups for thigh hypertrophy. Conversely, the study performed by Joy et al. [18] reported a 22.2 % increase in rectus femoris cross sectional area for the PA group that was significantly greater (*p* = 0.02) than the 13.3 % increase in rectus femoris cross sectional area for the PLA group.

In comparing the effects of PA on P, the results of this investigation were also different than those reported by Joy et al. [18]. Although no significant group x time interactions for P were observed in this study or in the study performed by Joy et al. [18], it should be noted that P increased by 4.2 % more in the MT group as compared to the PLA group in this investigation and that P increased by 0.5 % more in the PLA group as compared to the PA group in the study performed by Joy et al. [18]. Hoffman et al. [17] did not evaluate the effects of PA on P.

This investigation also examined more variables than previous researchers [17, 18]. Although the improvements in these variables with MT appear to be higher than in PLA, they did not reach statistical significance. The lack of significance observed in the performance variables may be largely contributed to the lack of specificity of training in the prescribed training protocol. Since the training protocol prescribed to the participants was a resistance program to gain strength/hypertrophy, these were the variables that were improved by PA.

Some of the more robust effects found in the present study as compared to previous studies [17, 18] could be attributed to the added proprietary blend of L-Leucine, HMB, and vitamin D3 that was provided to the experimental group in addition to the PA. Evidence has demonstrated that if essential amino acids or protein are ingested before or after a workout, the effect of muscle protein synthesis may be magnified [33]. L-Leucine alone has been suggested as an effective supplement in stimulating muscle protein synthesis, even if administered in low dosages [34].

Similar to L-Leucine, an L-Leucine metabolite known as HMB has been shown to stimulate muscle protein synthesis to a similar extent as L-Leucine [20]. HMB has also been found to decrease muscle protein breakdown [20, 24]. In a study performed by Wilkinson et al. [20] investigating the effects of L-Leucine and HMB on human skeletal muscle protein anabolism, it was demonstrated that orally consumed HMB showed fast bioavailability in plasma and muscle and, similarly to L-Leucine, stimulated muscle protein synthesis (+70 % for HMB vs. +110 % for L-Leucine). The study also reported that HMB and L-Leucine both increased mTOR signaling; however, this was more pronounced with Leucine [20]. HMB consumption also reduced muscle protein breakdown by 57 % in an insulin-independent manner [20].

The addition of vitamin D3 to the active ingredient in this study could also potentially contribute to some of the findings. Although systemic review articles have concluded there are conflicting findings on the effect of vitamin D3 on strength [35, 36], it has been suggested that sports dietitians and physicians routinely assess vitamin D status and make recommendations to help athletes achieve a serum 25 (OH) D concentration of ≥ 32 and preferably ≥ 40 ng∙mL [37] as vitamin D3 may improve athletic performance in vitamin D-deficient athletes [38]. Although most studies have looked at the effects of vitamin D3 in the elderly, a study performed on vitamin D3 deficient healthy young adults reported a significant difference between treatment and control groups in grip strength (*p* < 0.001) and calf strength (*p* = 0.04) [39].

Collectively, it is probable that the combination of PA, L-Leucine, HMB, and vitamin D3 used as the active ingredients in this study work synergistically in order to improve LBM and strength beyond the impact of the training program itself. A study recently reported that vitamin D3 has been shown enhance the stimulating effect of L-Leucine and insulin on protein synthesis [28]. Similarly, another study postulated that L-Leucine and HMB enhance muscle anabolism by increasing muscle protein synthesis and reducing muscle protein breakdown through either a different and/or additional mechanism(s) [20]. Furthermore, it has been shown that an increase in intracellular levels of PA via a mechanical stimulus such as weight training and ingestion of exogenous sources of PA can promote the activation of mTOR signaling through different pathways that may collectively contribute to a larger effect of mTOR signaling [7–9]. Thus, the combination of an intense hypertrophy/strength goal oriented training program in conjunction with the ingestion PA, L-Leucine, HMB, and vitamin D3 appears to collectively improve LBM and strength to a greater extent than a hypertrophy/strength training program alone or a hypertrophy/strength training program plus PA alone.

Although the main active ingredient in this study was PA, the proprietary blend of L-Leucine, HMB, and vitamin D3 that was provided as the supplement to the experimental group could be viewed as a limitation as it is difficult to isolate the effects on LBM and strength of each ingredient alone. A second limitation to this study is the lack of participants that completed the study. A larger sample might have led to more statistically significant results. Finally, the lack of a specific training stimulus for the participants to improve VJ, AG, muscular endurance, and P in order to truly assess the effects of MT on these performance variables limited changes in these variables.

Future research on PA and MT is needed to further investigate its efficacy. It is possible that a training program that incorporates specific training for improvements in VJ, AG, muscular endurance, and P output on a cycle ergometer in conjunction with PA (as opposed to a PLA) could significantly improve these performance variables. Furthermore, PA could potentially improve body composition to a greater extent than was observed in these studies. Although diet was controlled in two of the three studies, all participants were provided with an isocaloric diet instead of a hypocaloric diet; it is unknown what would happen if the subjects were on a hypocaloric diet in regards to LBM and FM. Future research on PA could also focus the mechanism of action of the supplement, its effect on other populations (i.e. elderly, highly trained), the absorption profile of orally administered PA, and the safety of PA ingestion. Additionally, future research could investigate if the ingestion of PA, L-Leucine, HMB, and vitamin D3 in specific dosages are collectively more effective at improving LBM and strength than these supplements independently.

## Conclusions

MT significantly increased maximum LP strength, BP strength, and LBM as compared to PLA. Although a trend was noted in improvements of AG, TMM, VJ, muscular endurance, P, and FM, the changes observed in the MT group compared to the PLA group were not statistically significant. The findings of this investigation further confirm the studies performed on the potential efficacy of PA for improving lower body strength, upper body strength, and lean body mass.

## Availability of data

Raw data can for this manuscript can be found at https://osf.io/6zecp/.

## Abbreviations

AG, agility; ANOVA, analysis of variance; BP, bench press; cGMP, current good manufacturing practices; FM, fat mass; HMB, Beta-Hydroxy-Beta-Methylbutyrate; HPLC, high pressure liquid chromatography; LBM, lean body mass; LP, leg Press; MT, MaxxTOR; mTOR, mammalian target of rapamycin; P, power; PA, phosphatidic acid; PLA, placebo; PU, push ups; TMM, thigh muscle mass; VJ, vertical jump
